# Human T-cell Leukemia Virus Type 1 Molecular Variants, Vanuatu, Melanesia

**DOI:** 10.3201/eid1105.041015

**Published:** 2005-05

**Authors:** Olivier Cassar, Corinne Capuano, Laurent Meertens, Eliane Chungue, Antoine Gessain

**Affiliations:** * Institut Pasteur de Nouvelle-Calédonie, Nouméa, France;; † Institut Pasteur, Paris, France;; ‡ World Health Organization, Port-Vila, Vanuatu

**Keywords:** molecular epidemiology, Human retrovirus, HTLV-1, Melanesia, Vanuatu

## Abstract

Four of 391 Ni-Vanuatu women were infected with variants of human T-cell leukemia virus type 1 (HTLV-1) Melanesian subtype C. These strains had *env* nucleotide sequences ≈99% similar to each other and diverging from the main molecular subtypes of HTLV-1 by 6% to 9%. These strains were likely introduced during ancient human population movements in Melanesia.

Human T-cell leukemia virus type 1 (HTLV-1), a human oncoretrovirus, is the etiologic agent of adult T-cell leukemia and of tropical spastic paraparesis/HTLV-1–associated myelopathy. Molecular epidemiologic studies have shown HTLV-1 proviruses to be remarkably stable genetically. The low levels of genetic drift in this virus have been used as a means for monitoring viral transmission and the movement of ancient human populations (1,2). The few nucleotide substitutions observed in HTLV-1 strains are specific to the geographic origin of the patient and are unrelated to viral pathology (1,2). Four major geographic HTLV-1 subtypes have been described: subtype A, cosmopolitan (1,2); subtype B, central African; subtype C, Melanesian (3–6); and subtype D, present in central Africa, mainly in pygmies.

Previous reports have indicated that HTLV-1 is endemic in some remote or ancient populations in Melanesia (3–14). These populations include a small number of tribes from Papua New Guinea (especially the Hagahai people) (5) and some inhabitants of the Solomon Islands (7). Evidence of HTLV-1 infection has also been found in some aboriginal groups from Australia (8). Rare cases of adult T-cell leukemia and tropical spastic paraparesis/HTLV-1–associated myelopathy have also been described in these populations (9).

Genetic characterization of the few available Melanesian HTLV-1 strains has indicated that these HTLV-1 strains are the most divergent, constituting molecular subtype C (also called Melanesian subtype [3,4, 6,10]) in phylogenetic analyses. The discovery of such divergent variants has increased our understanding of the migration of HTLV-1–infected populations throughout the Pacific region. Furthermore, 1 of the calibration methods frequently used, in phylogenetic analyses, to estimate a time scale for the evolution of HTLV and simian T-cell leukemia virus (STLV) appears to coincide with the first human migrations to Melanesia and Australia 40,000–60,000 years ago (2).

We carried out a large serologic and molecular study to determine the prevalence of HTLV-1 and associated diseases in the Vanuatu Archipelago. Vanuatu, formerly known as the New Hebrides, is a Y-shaped archipelago made up of ≈80 islands. It is located in Melanesia, in the South Pacific region, northeast of Australia and south of the Solomon Islands. Vanuatu has a population of ≈200,000 inhabitants, most of whom (95%) are of Melanesian origin and are known as the Ni-Vanuatu.

ecade ago and were not based on stringent serologic criteria (11–14). No molecular characterization data are available for HTLV-1 from this area. The main goals of this study were to evaluate the situation concerning HTLV-1 infection in a remote Ni-Vanuatu population by using stringent serologic criteria for Western blotting and molecular characterization of the viruses.

## The Study

In February 2002, we recruited 391 women during a clinical survey for sexually transmitted diseases in various remote rural communities of western Ambae Island in the Penama Province of the Vanuatu Archipelago. Ambae Island, also known as Aoba, has a population of ≈9,500. The women participating in this survey were offered a complete clinical examination, with Papanicolaou test analysis for all women >25 years of age. For each participant, we obtained plasma and buffy coats from 5 mL of blood obtained by venipuncture. The blood samples were rapidly transferred to Institut Pasteur de Nouvelle-Calédonie, where plasma and buffy coats were isolated, frozen, and stored (at –80°C) until HTLV screening. Informed consent was obtained from each woman participating in the field survey. This study was approved by the Ministry of Health of the Republic of Vanuatu and was supported by the Vanuatu Family Health Association, a local nongovernmental organization. Samples were taken from 391 women (mean age 36 years, range 16–82 years) with the following stratification by age: 11.2% from women 15–24 years of age, 28.4% from women 25–34 years of age, 31.2% from women 35–44 years of age, 17.4% from women 45–54 years of age, and 11.8% from women ≥55 years of age.

Plasma HTLV-1 antibodies were detected by enzyme-linked immunosorbent assay (ELISA) (HTLV-I+II, Abbott-Murex, Kent, United Kingdom) with Western blot (HTLV-I/II Blot 2.4, Diagnostic Biotechnology, Singapore) used for confirmation. On Western blot, plasma samples were considered HTLV-1–positive if they reacted to the 2 Gag proteins (p19 and p24) and both *env*-encoded glycoproteins: the HTLV-1–specific recombinant gp46-I peptide (MTA-1) and the specific HTLV-1/HTLV-2 recombinant GD 21 protein. Plasma samples were considered negative when no band were shown and indeterminate when partially reactive (15,16).

**Figure 1:**
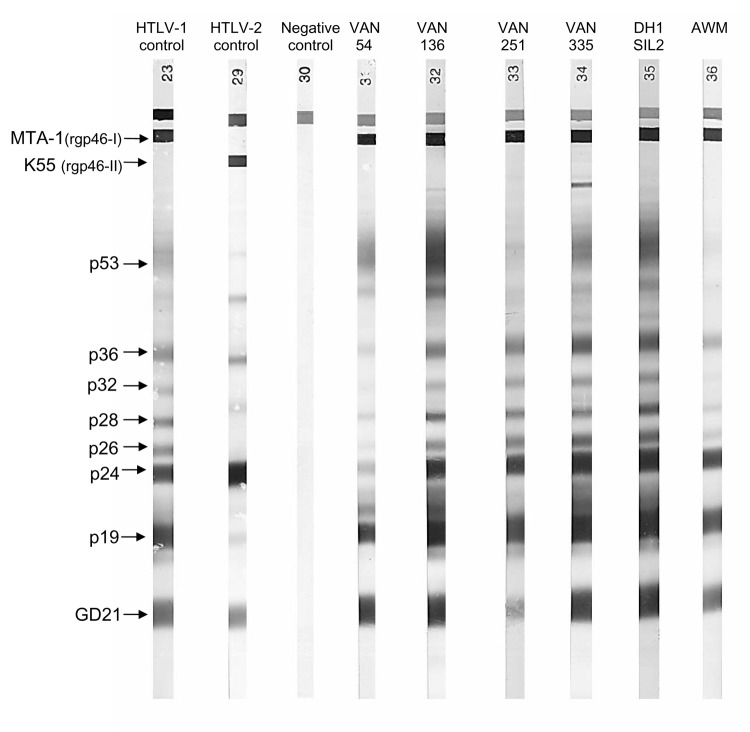
Representative seroreactivity pattern on Western blot that contains a recombinant GD 21 (common to human T-cell leukemia virus type 1 [HTLV-1] and HTLV-2) and 2 synthetic peptides specific for HTLV-1 (MTA-1) and HTLV-2 (K55). Lane 1, HTLV-1–positive control; lane 2, HTLV-2–positive control; lane 3, HTLV-1/2 negative control; lane 4–9, 6 plasma samples from the HTLV-1–positive women of Ambae Island displaying strong reactivity to GD 21 (faint band for VAN 251), to p19 and p24 (faint band for VAN 54), p26, p28, p32, p36 (faint bands for VAN 54 and AWM), and to MTA-1.

Forty-nine of the 391 plasma samples studied tested positive or borderline by ELISA, and 4 of these samples displayed full reactivity on Western blot (Figure 1). One sample also displayed a typical HTLV *gag*-indeterminate profile (16), and 6 displayed weak reactivity (19 or GD 21 bands). The 4 plasma samples testing positive by Western blot had higher immunofluorescence assay titers on MT2 (HTLV-1) cells than on C19 (HTLV-2) cells and high particle agglutination titers (Table 1). We carried out a second serologic survey on 64 members of the families of the 4 women seropositive for HTLV-1. This survey identified 2 more infected women; 1 was the mother of an index patient, and the other was the sister-in-law of another index patient (Table 1). These results confirm the circulation of HTLV-1 in this population.

**Table 1:** Human T-cell leukemia virus type 1 (HTLV-1) antibody titers and molecular screening results for HTLV-1–seropositive women from Ambae Island, Vanuatu Archipelago*

Virus strain	Age (y)	PA titers	IFA titers		WB pattern	PCR		
			MT 2	C 19		3´ LTR	5´ LTR	*env*
VAN 54	45	1/2,048	1/320	1/80	HTLV-I	+	+	+
VAN 136	36	1/8,192	1/1,280	1/320	HTLV-I	+	+	+
VAN 251	42	1/1,024	1/40	<1/20	HTLV-I	+	+	+
VAN 335	42	1/4,096	1/1,280	1/160	HTLV-I	+	+	+
DH1SIL2 (sister-in-law of VAN 335)	56	1/8,192	1/2,560	1/320	HTLV-I	NA	NA	NA
AWM (mother of VAN 54)	63	1/1,024	1/160	1/40	HTLV-I	NA	NA	NA

*PA, particle agglutination; IFA, immunofluorescence assay; WB, Western blot; PCR, polymerase chain reaction; LTR, long terminal repeat; NA, DNA not available.

High molecular-weight DNA was extracted from buffy coats from the 4 HTLV-1–seropositive women, 5 HTLV-1–seronegative persons, and 6 others with indeterminate Western blot results, by using the QIAamp DNA Blood Mini Kit (Qiagen GmbH, Hilden, Germany). The 15 DNA samples studied were subjected to polymerase chain reaction with primers specific for the human β-globin gene to check that cellular DNA was amplifiable for all samples (17). We then subjected DNA samples to 2 series of polymerase chain reaction to obtain the complete long terminal repeat (LTR) (755 bp) and a 522-bp region of the *env* gene as previously described (18). Fragments of the appropriate size were amplified for the 4 HTLV-1–seropositive women, whereas the other 11 samples yielded negative results. The amplified products were cloned and sequenced, and phylogenetic studies were performed as previously described (18). Both the complete LTR and the 522-bp *env* fragment were obtained for the 4 HTLV-1–seropositive women.

## Conclusions

The gp21 gene sequences of the 4 HTLV-1 strains involved were almost identical (99.6%–99.8 % nucleotide similarity) and were very similar to those of Melanesian strains. These strains were closely related (99.4%) to certain strains from Solomon Islanders (Mel 4, 8) but were only 97.1%–98.3% similar to strains from Papua New Guinea residents (Mel 2, 7) and from Australian aborigines (MSHR-1), respectively. Finally, the sequences of these new strains diverged from those of HTLV-1 strains from the 3 other main molecular subtypes (A, B, D) by 6% to 9%.

The 4 new HTLV-1 LTR sequences were also very closely related (98%–100% nucleotide similarity). They displayed 2% nucleotide divergence from Mel 5 (from a Solomon Islander), the only available LTR from all the HTLV-1 subtype C strains. However, they also displayed up to 11% nucleotide divergence from HTLV-1 strains from other molecular subtypes.

**Figure 2:**
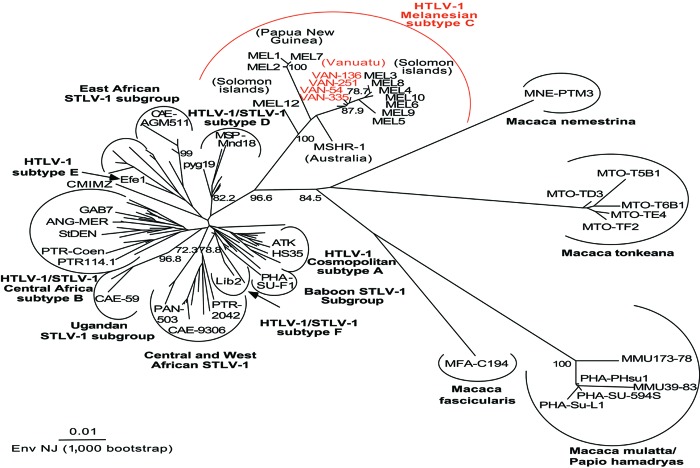
Unrooted phylogenetic tree generated by the neighbor-joining method by using the 522-bp fragment of the *env* gene. Distance matrices were generated with the DNADIST program, using the Kimura 2-parameter method and 5.65 as the transition/transversion ratio. Bootstrap analysis was carried out with 1,000 datasets. The values on the branches indicate frequencies of occurrence for 1,000 trees. The 4 new human T-cell leukemia virus type 1 (HTLV-1) sequences (VAN 54, VAN 136, VAN 251, VAN 335; GenBank accession nos. AY549879, AY549880, AY549881, AY549882) were analyzed with 126 HTLV-1/simian T-cell leukemia virus type 1 (STLV-1) sequences available from the GenBank database. The branch lengths are proportional to the evolutionary distance between the taxa.

**Figure A1:**
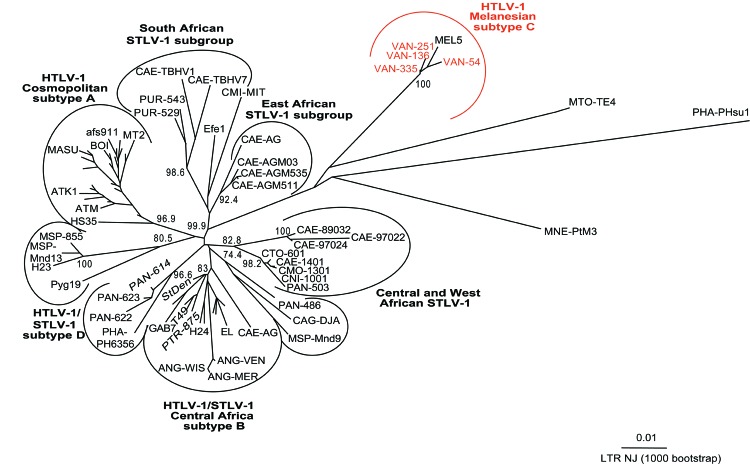
Unrooted phylogenetic tree generated by the neighbor-joining method by using the complete fragment of the long terminal repeat (LTR) (755 bp). Distance matrices were generated with the DNADIST program, using the Kimura 2-parameter method and 5.17 as the transition/transversion ratio. Bootstrap analysis was carried out with 1,000 datasets. The values on the branches indicate frequencies of occurrence for 1,000 trees. The 4 new human T-cell leukemia virus type 1 (HTLV-1) sequences (VAN 54, VAN 136, VAN 251, VAN 335; GenBank Accession nos. AY549875, AY549876, AY549877, AY549878) were analyzed with 75 HTLV-1/STLV-1 sequences available from the GenBank database. The branch lengths are proportional to the evolutionary distance between the taxa.

Phylogenetic analyses were performed on all the available *env* and LTR HTLV-1 sequences from Melanesia, and on several representatives of HTLV-1 and STLV-1 strains from the various subtypes/subgroups as described (18), by the neighbor-joining (NJ) method. Similar tree topologies were obtained for both genomic regions (Figure 2 and Figure A1). Analyses of these trees confirmed that the 4 novel Vanuatu HTLV-1 strains were closely related to all available HTLV-1 subtype C strains (Table 2). Indeed, in the *env* analysis, which included 71 HTLV-1 strains (including 12 Melanesian strains and 1 from an Australian aborigine, Table 2) and 55 STLV-1 strains, the 4 new HTLV-1 strains clustered with subtype C (Figure 2). This subtype only includes strains from Australia, Papua New Guinea, the Solomon Islands, and Vanuatu. Within this clade are at least 2 subgroups, strongly supported phylogenetically: 1 comprises the Vanuatu strains and most of the strains from the Solomon Islands (bootstrap values of 88%), and the other comprises the 3 isolates from Papua New Guinea (the Hagahai population), with a bootstrap value of 100%. Two other unique and divergent strains, the only strain available from an Australian aborigine (MSHR-1) and the other from a Solomon Islander (Mel-12), may represent prototypes of 2 other clades within the Melanesian subtype C.

**Table 2:** Epidemiologic data and GenBank accession numbers of the human T-cell leukemia virus type 1 (HTLV-1) strains of the Melanesian subtype C

Country of origin	Age (y)	Sex	Birth	Residence	Clinical status	Virus name	*env* GenBank accession no.	LTR GenBank accession no.
Vanuatu	45	F	Ambae	Filakalaka	AC	HTLV-1 VAN 54	AY549879	AY549875
	36	F	Ambae	Ndui Ndui	AC	HTLV-1 VAN 136	AY549880	AY549876
	42	F	Ambae	Vinangwangwe	AC	HTLV-1 VAN 251	AY549881	AY549877
	42	F	Ambae	Lolobinanungwa	AC	HTLV-1 VAN 335	AY549882	AY549878
Papua New Guinea	21	M	Madang	Madang	AC	HTLV-1 MEL 1	L02533	NA
	60	F	Madang	Madang	AC	HTLV-1 MEL 2	M94197	NA
	31	M	Madang	Madang	AC	HTLV-1 MEL 7	U11576	NA
Solomon Islands	39	F	New Georgia	Guadalcanal	AC	HTLV-1 MEL 3	M94198	NA
	60	F	Guadalcanal	Guadalcanal	AC	HTLV-1 MEL 4	M94199	NA
	58	M	Guadalcanal	Guadalcanal	AC	HTLV-1 MEL 5	M94200	L02534
	38	M	Guadalcanal	Guadalcanal	TSP/HAM	HTLV-1 MEL 6	M93099	NA
	49	M	New Georgia	Guadalcanal	AC	HTLV-1 MEL 8	U11578	NA
	75	M	Rendova	Guadalcanal	AC	HTLV-1 MEL 9	U11580	NA
	13	F	Guadalcanal	Guadalcanal	AC	HTLV-1 MEL 10	U11566	NA
	42	F	Guadalcanal	Guadalcanal	AC	HTLV-1 MEL 11	U11568	NA
	60	F	Guadalcanal	Guadalcanal	AC	HTLV-1 MEL 12	U11570	NA
Australia	NA	NA	NA	NA	AC	HTLV-1 MSHR-1	M92818	NA

*LTR, long terminal repeat; F, female; M, male; AC, Asymptomatic carrier; TSP/HAM, tropical spastic paraparesis/HTLV-1–associated myelopathy; NA, not available.

In conclusion, we report, for the first time, the presence of HTLV-1 infection in a Ni-Vanuatu population living in remote villages. We also demonstrate that the viruses infecting these Ni-Vanuatu persons are novel HTLV-1 molecular variants belonging to the Melanesian divergent C subtype. This finding suggests that these viruses were introduced into Vanuatu by ancient migrations of Melanesian populations. The first people to reach Santa Cruz, Banks, Vanuatu, and the Loyalties Islands ≈3,600 years ago seem to have been Austronesian speakers (19). Epidemiologic and clinical surveys are under way in this area to determine the extent of such retroviral infection and associated neurologic and hematologic diseases. In addition, studies of viral and mitochondrial/nuclear DNA are being conducted and should provide insight into the migrations of the first settlers and the origin, evolution, and modes of dissemination of such retroviruses.
